# A Comparative Study of the Registry System effect on Patients Satisfaction Rate in Two Emergency Department Settings

**DOI:** 10.30476/BEAT.2021.84704.1076

**Published:** 2021-07

**Authors:** Orhan Delice, Samad Shams Vahdati, Senol Arslan, Alireza Alireza, Hossein Hosseinifar, Faride Houshmand, Solomon Habtemariam, Aysa Rezabakhsh

**Affiliations:** 1 *Emergency Medicine Department, Regional Research and Education Hospital, Erzurum, Turkey*; 2 *Emergency Medicine Research Team, Tabriz University of Medical Sciences, Tabriz, Iran*; 3 *Evidence-Based Medicine Research Center, Tabriz University of Medical Sciences, Tabriz, Iran *; 4 *Pharmacognosy Research Laboratories & Herbal Analysis Services UK, University of Greenwich, Chatham-Maritime, Kent ME4 4TB, UK*; 5 *Cardiovascular Research Center, Tabriz University of Medical Sciences, Tabriz, Iran*

**Keywords:** Satisfactory rate, Emergency services, Patient admission, Health care quality

## Abstract

**Objective:**

To assess the patient’s satisfaction rate during two distinct registry procedures in the emergency department.

**Methods:**

A cross-sectional study was conducted in educational hospitals with a high volume of patient’s admission in Tabriz-Iran and Erzurum-Turkey. In this study, we used a Press Ganey questionnaire as a data collection tool that was filled out with patients or their companions before discharging or referred to other areas (wards). Finally, data were analyzed by using SPSS software version 16.

**Results:**

The included patients were from three-admission time courses includes morning, evening, and night shifts. The present study results indicated that the total satisfaction score was two scores higher than the classic one (*p*<0.001) in the model registry system. Furthermore, the findings of the current study interestingly showed a correlation between satisfaction rate and education level as well as patient’s location. Thus, patients with moderate education levels had a higher satisfaction rate in urban regions when compared with rural regions and higher/lower education levels (*p*=0.03).

**Conclusion:**

Patients’ satisfaction rate with multiple variables can be improved by designing an appropriate registry procedure.

## Introduction

The emergency department (ED) admission represents a significant proportion of hospital admission around the world. The ED challenges that affect the overall patients’ healthcare outcomes include overcrowding, delays in throughput, boarding of admitted patients, hospital longer stay, inaccessibility to appropriate hospital beds, and lost opportunities to access on-time patients care services [1, 2]. Admission delays in ED have also a socio-economic impact in addition to possible health impacts. Upon arrival to the ED, patients are prioritized individually based on the acute requirement of urgent medical intervention which is called “triage”. This is routinely performed by one of the expert hospital staff, according to the patient’s chief complaint, demographic characteristics, and vital signs. In this regard, the patient should be visited by a medical provider who makes the initial care plan and finally recommends a disposition. 

Emergency medical management is considered as an essential component of primary care and a professional discipline in the field of modern medicine [3]. The initial identification of a medical emergency and subsequent management will prevent irreversible outcomes, and unintended consequences that help the health care system to save the patient’s life [4]. On the other hand, a reliable registry system is essential to manage the record of both hospitalized and discharged patients. Hitherto, there are different management strategies related to ED, such as a long-term waiting caused by unreasonable factors, the unbalance operation of physicians and nurses, and so on [5]. Registration is one of the critical management issues in the ED setting [6]. The main purpose of the registration process is the reliable identification of the patient to ensure the information obtained following entering historical data in the Hospital Information System (HIS) for better diagnostic accuracy [7]. The second factor is timeliness which plays an important role to determine patients’ satisfaction. Hence, a lengthy registration process that affects patients’ safety and care must be avoided, in addition to frustration due to long waiting time in the ED [8]. The results of one survey study reported a low satisfaction rate by patient’s companions after spending more time in registration queues [9]. Recently, He *et al*., [5] designed an optimized registration process by using allocated resources based on system simulation to reduce waiting time in the emergency department of West China Hospital. Another research also demonstrated that alternative admission policies by different physicians improved the registry process and reduced waiting times [10]. To date, there is limited evidence regarding the satisfaction rate during the registry process. Therefore, in this study, we aimed to compare the satisfaction rate of central (classic) and local (model) registrations for timely triage to minimize pitfalls in admission services and enhance the expected satisfaction rate. Regarding the lack of documented clinical research, the results of this study could help to improve the management issues quality in emergency medicine and other health care systems in evaluating different registry implementation in Iran. 

## Materials and Methods

*Study Design and Patient Population*

Tabriz (the capital city of East Azerbaijan Province, in northwestern Iran) and Erzurum (a city in eastern Anatolia, Turkey), which are culturally and climatically similar, were used in this comparative study as they used different registry processes of ED. An analytic cross-sectional study was conducted on 462 patients who were referred to ED of both Imam-Reza and Bölge hospitals in Tabriz-Iran and Erzurum-Turkey, respectively with high volume admission and approximately 400 to 600 daily visits from April 2019 to August 2019. According to the calculation of sample size after the pilot study, a minimum of 110 patients were needed to be included. Meeting this requirement, 199 and 263 patients participated in Imam-Reza and Bölge ED, respectively. In this study, the survey research method was used to estimate the satisfaction rate of admitted subjects who were randomly assigned by Excel software in different time courses (morning, evening, and night) in the ED of both hospitals.

*Patient Satisfaction Questionnaire*

**Fig. 1:**
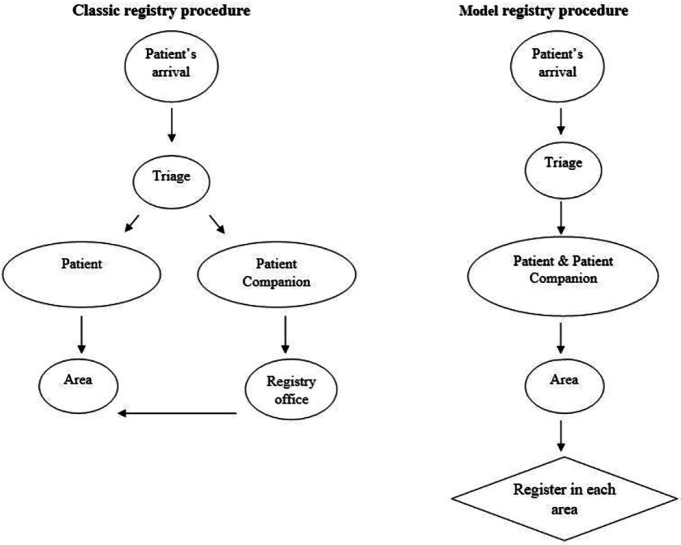
The algorithm of two different registry procedures. Left side explains the central registry system employed in the most ED, while, right side shows the local registration (model) in each area for timely triage with the aim to minimize delays in admission services and raising the expected satisfaction rate

In consideration of the study purpose, a survey was designed with some modifications based on a reliable questionnaire of the previous study [11]. The questionnaire comprised of different sections includes of the first part contained the general demographical questions (i.e. gender, age, location of living, and academic degree), which were analyzed qualitatively. The second part referred to the waiting time evaluation in different registry systems and was calculated quantitatively. The last part was also related to the main purpose and showed the patient’s satisfaction rate according to the achieved scores (supplementary I). By using a distinct registration system, namely model registration at Erzurum hospital, we precisely evaluated demographic characteristics, time-related parameters, and the efficiency of model registration. Therefore, the obtained results were compared with the conventional system used at Tabriz Imam-Reza hospital. The structured, pre-tested press Ganey questionnaire was filled out by one of the hospital workers during the face-to-face interview. To note, the subjects who were willing to participate in this study were interrupted the interview only to clarify a question if required, but they did not reveal any information about the questions. The inclusion criteria included all patients who referred to the ED and were triaged while the exclusion criteria consisted of all patients who quit the ED for any reason other than admission or discharge. Figure 1 precisely describes the different steps of the two registry systems.

*Statistical Analysis*

In the present study, the satisfaction score of all patients as well as waiting times for both classic and model registration systems were compared along with the demographic characteristics. The results were reported as percentage and mean±standard deviation (SD) for qualitative and quantitative variables, respectively. Qualitative variables were also analyzed between two different systems by the Pearson Chi-square test. Univariate analysis was used to compare the satisfaction score mean on the levels of independent variables by using independent t-test and analysis of variance (ANOVA). We also used Tukey’s test for post-hoc analysis where it was applicable. Linear regression model also fitted data to evaluate the factors that affected satisfaction score. Those variables which had a *p*-value less than 0.2 in univariate analysis were entered into the regression model. Our data were analyzed by using SPSS software version 16 (SPSS Inc., Chicago, IL, USA). The *p* values less than 0.05 were considered statistically significant.

## Results

Of 462 participants, 199 (43.1%) and 263 (56.9%) patients were admitted through the classic and model registry system, respectively. The patients’ mean age was 44.32±22.27 (the range between 0-94 years old). The demographic characteristics of all subjects were fully explained in the two different registry systems (Table 1). 

**Table 1:** Demographic Characteristics of All Admitted Patients

		**Registration Method n (%)**		***p*** **-value** ^a^
		**Classic**	**New**	
Gender	Male	110 (55.28%)	121 (46.01%)	0.04
	Female	89 (44.72%)	142 (53.99%)	
First Visit	Yes	96 (48.24%)	216 (87.1%)	<0.001
	No	103 (51.76%)	32 (12.9%)	
Who is Completing	Patient	25 (12.56%)	146 (55.51%)	<0.001
	Patient Companion	174 (87.44%)	117 (44.49%)	
Residential Place	Urban Area	89 (44.95%)	219 (83.59%)	<0.001
	Rural Area	109 (55.05%)	43 (16.41%)	
Admission Shift	Morning	77 (38.69%)	45 (17.24%)	<0.001
	Evening	45 (22.61%)	67 (25.67%)	
	Night	77 (38.69%)	149 (57.09%)	
Education	Uneducated	153 (76.88%)	122 (49.39%)	<0.001
	Diploma	32 (16.08%)	88 (35.63%)	
	Academic	14 (7.04%)	37 (14.98%)	

^a^ *p*-value calculated by Pearson Chi-square test

Our data displayed a significant difference between different admission shifts regarding the satisfactory score based on the one-way ANOVA analysis. Also, the results of the Tukey test were confirmed a difference between the satisfaction score mean of the morning and night shifts (*p*<0.001), as well as the evening and night shifts (*p*=0.022). However, we could not find any significant difference between the morning and evening shifts in terms of the average satisfaction score (*p*=0.227). We reported a remarkable difference between uneducated and diploma groups in regard of the educational level effects on the satisfactory rate (Table 2**,** *p*=0.028). Consequently, our results revealed that the satisfaction score total average increased considerably in the model registry system when compared to the classic system (16.41±3.23 vs. 14.66±3.9; *p*<0.001).

**Table 2:** Results of Univariate Analysis for Comparison of Satisfaction Score in Levels of in Dependent Variables

		**N**	**Mean±SD**	***p*** **-value** ^a^
Method	Classic	197	14.66±3.9	<0.001
	New	259	16.41±3.23	
Who is Completing	Patient	170	16.39±3.27	0.001
	Another one	286	15.22±3.78	
Admission shift	Morning^ d^	119	16.77±3.49	<0.001^b^
	Evening^e^	112	16±3.55	
	Night^de^	223	14.90±3.61	
First Visit	Yes	308	15.59±3.57	0.91
	No	134	15.54±3.81	
Gender	Male	226	15.36±3.75	0.08
	Female	230	15.95±3.51	
Location	Urban area	303	16±3.53	0.003
	Rural area	151	14.93±3.76	
Education	Uneducated^c^	271	15.27±3.71	0.03^b^
	Diploma^c^	119	16.3±3.35	
	Academic	51	15.67±3.86	

^a^ *t*-independent test; ^b^One-way ANOVA; ^c^ Significant due to Post-Hoc Test; ^d^ Significant due to Post-Hoc Test; ^d^ Significant due to Post-Hoc Test

Following the Pearson’s correlation coefficient, the relationship between satisfaction score and age (r=-0.18), time for the first visit (r=-0.33), time for any area transferring (r=-0.26), time for register (r=-0.22), and time for decision (r=-0.17) were statistically significant (*p*<0.001). The analysis of different time courses of the first visit, area transferring, time for registration, and a final decision was performed to assess the quality of both registry systems. According to our findings, the requested time for the first visit and area transferring was significantly prolonged in the classic registry system (Table 3, *p*<0.001). Although the times of registration and the final decision were extended longer in the model method, the satisfaction score of the model registry system was approximately two scores higher which directly refers to the quality of registration (Table 3, *p*<0.001). 

**Table 3.** Comparison of Different Time Courses for Satisfaction Score of the Registry Procedure

**Method**		**N**	**Mean±SD**	***p*** **-value**
Time for First Visit	Classic	199	18.02±25.88	<0.001
	Model	263	7.55±8.81	
Time for Any Area Transfer	Classic	199	7.13±8.85	<0.001
	Model	258	4.03±3.7	
Time for Register	Classic	199	2.61±13.95	0.028
	Model	263	4.9±5.14	
Time for Final Decision	Classic	199	31.82±51.31	<0.001
	Model	245	80.83±116.08	

Moreover, the multivariable linear regression analysis was performed to calculate the impact of independent variables on satisfaction scores. According to the results of regression modeling, some variables significantly affected the average variations of satisfaction score including routs of a registry, first admission, the first visit time, the registration time, and the time for final decision (Table 4, *p*<0.001). As shown in Table 4, the average satisfaction score in patients admitted through the model registry, 1.8 units was higher than the classic one (β=1.80, *p*<0.001). Besides, the average satisfaction score for the patients who were visited for the first time was 0.85 score higher than the patients referred more than once (Table 4, β=0.85, *p*<0.001). Linear regression modeling also determined that a prolonged time for the first visit, register, and final decision leads to the reduction of satisfaction score. 

**Table 4:** Predictors of patient’s satisfaction

		**β (SE)**	**t-value**	**95% CI**	***p*** **-value**
**Registration Method**	Classic	**Ref**			
	Model	1.8(0.43)	4.21	(0.96 to 2.64)	<0.001
**Who Is Completing**	Patient	**Ref**			
	Patient Companion	0.16(0.39)	0.418	(-0.6 to 0.92)	0.676
**First Visit**	Yes	**Ref**			
	No	-0.85(0.2)	-4.234	(-1.24 to -0.46)	<0.001
**Gender**	Male	**Ref**			
	Female	0.07(0.31)	0.237	(-0.54 to 0.68)	0.813
**Residential Place**	Urban Area	**Ref**			
	Rural area	0.05(0.37)	0.128	(-0.68 to 0.78)	0.898
**Education**	Uneducated	**Ref**			
	Diploma	0.26(0.41)	0.638	(-0.54 to 1.06)	0.524
	Academic	0.09(0.52)	0.173	(-0.93 to 1.11)	0.863
Age		-0.01(0.01)	-0.825	(-0.03 to 0.01)	0.41
Time for First Visit		-0.05(0.01)	-5.632	(-0.07 to -0.03)	<0.001
Time for Any Area Transfer		-0.05(0.02)	-1.882	(-0.09 to -0.01)	0.061
Time for Register		-0.06(0.02)	-3.889	(-0.1 to -0.02)	<0.001
Time for Decision		-0.01(0.002)	-3.591	(-0.01 to -0.006)	<0.001

The results of the satisfaction score of inter-group in compare with each system are shown in Table 5. Based on independent *t*-test analysis, the statistically significant differences were observed in both classic and model registry systems related to some variables such as time of the visit and those who were completing (Table 5, *p*<0 .05). However, the time of admission for both registry systems was significantly different, and the highest satisfaction rate was observed in the morning time course (Table 5, *p*<0.001). 

**Table 5:** Comparison of Satisfaction Score Regarding to Independent Variables in Two Registration systems

		**N**	**Classic Registration**	***p*** **-value**	**N**	**New Registration**	***p*** **-value**
			**Mean±SD**			**Mean±SD**	
Who is Completing	Patient	25	14.00±3.57	0.36	145	16.8±3.04	0.03
	Another one	172	14.76±3.95		114	15.92±3.41	
Admission Shift	Morning	75	16.05±3.90	<0.001	44	18.00±2.20	<0.001
	Evening	45	13.38±3.44		67	17.76±2.33	
	Night	77	14.06±3.79		146	15.34±3.44	
First Visit	Yes	95	14.07±3.86	0.04	213	16.26±3.23	0.59
	No	102	15.22±3.88		32	16.59±3.43	
Gender	Male	108	14.33±3.99	0.19	118	16.31±3.26	0.62
	Female	89	15.07±3.78		141	16.5±3.22	
Location	Urban area	88	14.98±4.14	0.3	215	16.42±3.17	0.79
	Rural area	108	14.40±3.72		43	16.28±3.58	
Education	Uneducated	151	14.6±3.83	0.51	120	16.12±3.40	0.47
	Diploma	32	15.28±3.79		87	16.68±3.12	
	Academic	14	13.93±4.98		37	16.32±3.18	

## Discussion

Patients’ satisfaction rate identifies the percentage of patients who are satisfied and pleased with their healthcare services [12, 13]. As a measure of assessing health care quality, patient satisfaction gives new insights into the various aspects of medicine including the effectiveness of care services [11]. Patients’ satisfaction has recently gained notoriety in various medical centers as an important factor when delivering any kind of in-hospital care service [14]. In a patient-centered health care system, patients are expected to demand and expect high-level and transparent healthcare services from their providers [15]. Therefore, the assessment of patient’s satisfaction rate is thought to be one of the imperative indicators that can be measured in a self-report study through a questionnaire [15]. Additionally, a fragmented and inefficient admission process may result in delay in transition and restricts that flows both in- and outpatients [16]. In this regard, our results showed that ED registration could improve patient satisfaction rate in each area and unmet needs in case of the health care services improvement to compare with the central admission system. In line with our results, Soleimanpour *et al*., [11] showed that the rate of general satisfaction was calculated about 34.9% in emergency medicine. However, no statistically significant differences were shown between gender and satisfaction rate. In contrast, McKinley *et al*. demonstrated that satisfaction rate directly correlated with gender, age, and social status [17-19]. Based on our findings, a rational correlation was observed between satisfaction score and living location in the model registry system. In other words, the satisfaction rate in most of the urban patients was significantly higher than in rural regions (*p*=0.003). In this respect, some reasons can be mentioned for rural individuals such as lack of accessibility to facilities or accommodation problems [20, 21]. It should be mentioned that the satisfaction rate with moderate level of education was higher than the patients with higher/lower education levels (*p*=0.003) in patients from urban regions. This can be related to a more realistic point of view and a rational level of their expectation. Finally, it would be better to organize a throughput chart that consists triage classification rules in each level with a clear illustration of the patient’s companion presence necessity. This leads to an increase patient’s orientation and satisfaction rate as well as reasonable expectations during the triage and registration process in ED. 

One of the most important criteria in health care systems is providing a high-quality care service. In this study, we tended to compare two applied registry procedures and evaluated the rate of satisfaction in the admitted patients to improve patient flow, particularly in ED. Consequently, our findings clarified that the model registry system has more priorities and higher satisfactory scores in comparison to the conventional setting. 

## Limitation

The limitations of this study are includes the following: first, this study was conducted in two separate educational hospitals in different regions where the effects of admission policies were framed in the context of training mission and cannot be generalized to other hospitals. Therefore, the outcomes of the current study may be confined to only academic health care settings. The second limitation relates to the sample size and therefore, we suggest to design large-scale research for future studies.

## Abbreviations

ED: Emergency Department; HIS: Hospital Information System; SPSS: Statistical Package for the Social Sciences; ANOVA: Analysis of Variance; SD: Standard Deviation

## Availability of data and materials:

All data generated or analyzed during this study are included in this published article.
